# Emergence of antibiotic resistance in immunocompromised host populations: A case study of emerging antibiotic resistant tuberculosis in AIDS patients

**DOI:** 10.1371/journal.pone.0212969

**Published:** 2019-02-28

**Authors:** Ashley A. DeNegre, Martial L. Ndeffo Mbah, Kellen Myers, Nina H. Fefferman

**Affiliations:** 1 Command, Control, and Interoperability Center for Advanced Data Analysis, Rutgers University, New Brunswick, New Jersey, United States of America; 2 Department of Ecology, Evolution, & Natural Resources, Rutgers University, New Brunswick, New Jersey, United States of America; 3 Yale School of Public Health, New Haven, Connecticut, United States of America; 4 Department of Ecology & Evolutionary Biology, University of Tennessee, Knoxville, Tennessee, United States of America; Nankai University, CHINA

## Abstract

**Objective:**

The evolution of antibiotic resistance is far outpacing the development of new antibiotics, causing global public health concern about infections that will increasingly be unresponsive to antimicrobials. This risk of emerging antibiotic resistance may be meaningfully altered in highly AIDS-immunocompromised populations. Such populations fundamentally alter the bacterial evolutionary landscape in two ways, which we seek to model and analyze. First, widespread, population-level immunoincompetence creates a novel host environment with disrupted selective pressures. Second, within AIDS-prevalent populations, the recommendation that antibiotics be taken to treat and prevent opportunistic infection raises the risk of selection for drug-resistant pathogens.

**Design:**

To determine the impact of HIV/AIDS on the emergence of antibiotic resistance–specifically in the developing world where high prevalence and economic challenges complicate disease management.

**Methods:**

We present an SEIR epidemiological model of bacterial infection, and parametrize it to capture HIV/AIDS-attributable emergence of resistance under conditions of both high and low HIV/AIDS prevalence.

**Results:**

We demonstrate that HIV/AIDS-immunocompromised hosts can be responsible for a disproportionately greater contribution to emergence of resistance than would be expected based on population-wide HIV/AIDS prevalence alone.

**Conclusions:**

As such, the AIDS-immunocompromised have the potential become wellsprings of novel, resistant, opportunistic pathogen strains that can propagate into the broader global community. We discuss how public health policies for HIV/AIDS management can shape the evolutionary environment for opportunistic bacterial infections.

## Introduction

The rapid emergence of antibiotic resistant microbes represents a worldwide health risk since development of antibiotics is being outpaced by the evolution of resistance. [1] Factors contributing to resistance include prescribing habits of health professionals, antibiotic policy-making decisions, drug availability, and sociocultural beliefs regarding the necessity of antibiotics. [2] Regardless of the drivers of emergence, the result is the same: antibiotic-resistant infections. We have seen the dangers of drug resistance exemplified in bacterial pathogens such as *Escherichia coli*, *Streptococcus pneumoniae*, *Mycobacterium tuberculosis*, *Clostridium difficile*, and *Staphylococcus aureus*, each of which includes strains unresponsive to at least one antimicrobial agent. [2, 3]

We expect the risk of emergence to be magnified in regions where selection for drug-resistant pathogens is particularly high. Thus, we consider the potential for Human Immunodeficiency Virus- (HIV-) and Autoimmune Deficiency Syndrome- (AIDS-)prevalent populations to serve as hotbeds of emerging resistance. One of the most important tools in opportunistic infection management among the AIDS-immunocompromised is a constant regimen of antibiotics. [4] However, the use of antibiotics–even appropriately–exerts a strong selective pressure upon drug-sensitive pathogens. [5] Therefore, in highly HIV/AIDS-prevalent populations (>25% prevalence), [6] resistance develops and proliferates quickly [7] as constant use of antibiotics advances the emergence and maintenance of drug-resistant microbes. [8] This effect is compounded under conditions of limited drug availability and/or non-adherence to antibiotic protocols. [5] Under these circumstances partially resistant strains benefit from increased probability of survival, which also increases their chance of evolving greater resistance. Further, while antibiotics usually act in concert with the host’s immune system to combat infection, [9] AIDS-immunocompromised hosts lack the additional selective pressure imposed by immunocompetence against all pathogen replication. Even usually antibiotic-sensitive strains may therefore be able to survive ‘normal’ antibiotic doses longer in these patients. This effect can bolster their potential to survive long enough to increase resistance by mutation or horizontal gene transfer. [10, 11]

For these reasons, populations with a high prevalence of active AIDS cases represent novel environments with unique selective pressures leading to potentially drastically different probabilities of emergence of antibiotic resistance, relative to those expected in an immunocompetent population. This is particularly concerning in resource-poor regions where access to full courses of antibiotics may be limited by drug availability and economic constraints. [12] (We note that antibiotic adherence can also be undermined–and selection for resistance increased–by excessive availability of antimicrobials that can often be purchased without a prescription. [13]) If AIDS-prevalent regions are serving as wellsprings of drug resistance, emergence of resistance is not limited to just those areas. The movement of hosts who are either actively infective or harboring resistant, but currently benign, microbial strains can create a global health threat. [14] As the debate continues over how to allocate antibiotics so as to minimize the emergence and propagation of resistance, [11] policy decisions must account for resistant microbial strains spreading from one region to another via host migration. [15]

To demonstrate the potential impact of AIDS prevalence on the likelihood of emergence of antibiotic resistance in the developing world, we present an SEIR (susceptible, exposed, infective, recovered) compartmental model.[16] We parameterize this model to reflect conditions in two resource-limited communities, one with 27.4% HIV/AIDS prevalence (Swaziland), [6] and one with 0.46% HIV/AIDS prevalence (Indonesia). [17] In addition to the potential for non-adherence that is created by resource limitations, we have chosen to focus on the developing world for two reasons. First, despite the growing availability of antiretrovirals, developing nations remain at risk for an increase in the prevalence of HIV/AIDS (and, therefore, AIDS-defining illnesses). [18] Second, the growing incidence of nosocomial infections indicates that resistance will arise rapidly within hospital settings in these regions. [5] The combination of these factors suggests that AIDS-prevalent host populations in the developing world may ultimately be responsible for a disproportionate number of resistant infections, which presents a significant worldwide health risk to both immunocompetent and AIDS-immunocompromised individuals.

## Methods

Fully and functionally immunocompetent hosts can still contribute to antibiotic resistance. Thus, we use our model to quantify the relative contribution to the emergence of resistance from both fully immunocompetent and HIV/AIDS patients (HIV/AIDS+) receiving highly active antiretroviral treatment (HAART+). We also compare the actual contribution to emergence attributable to AIDS-immunocompromised hosts to that which would be expected based on AIDS prevalence alone. We do so by computationally reducing HIV/AIDS prevalence to zero in both countries and then measuring the magnitude of the corresponding decreases in infection prevalence and total emergence. Finally, holding antibiotic adherence constant among infectives, we calculate total emergence as a function of gradually increasing HIV/AIDS prevalence.

### Mathematical model

Our model examines the relative rate of emergence of antibiotic resistance in populations whose collective immunosuppression and prescribed antibiotic use patterns disrupt the selective pressures typically exerted on bacterial pathogens by host immune function and medically recommended antibiotic-taking behavior.

The vast difference in HIV/AIDS prevalence that exists between Indonesia (0.46%) and Swaziland (27.4%) [6, 17] suggests that there is a significant difference in the proportion of each population that is actively recommended to be taking antibiotics at any time.

**Fig 1:**
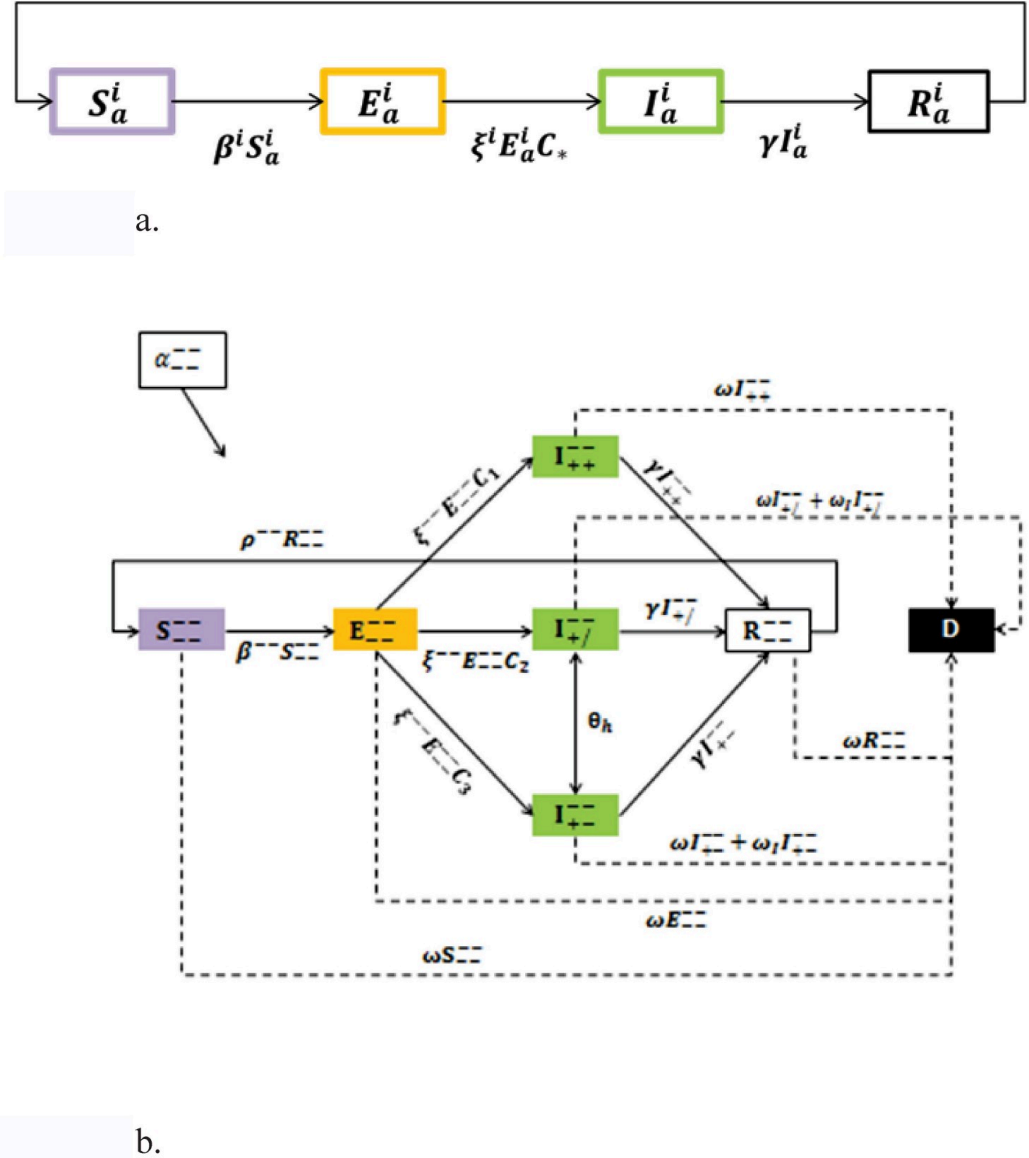
SEIR transmission dynamics. 1a. Basic SEIR model (assuming a closed system), wherein health status changes from susceptible to exposed at a rate of β, from exposed to infectious at a rate of ζ, and infectious to recovered at a rate of γ. The super- and subscripts “i” and “a” are used generically to demonstrate that there are many possible host health outcomes, depending on the combination of immune status and antibiotic-taking behavior. 1b. Transmission dynamics specific to our model; we note all possible progressions for an HIV/AIDS- host that contracts a bacterial pathogen.

We defined our population according to four descriptors: immune (HIV/AIDS) status, HAART adherence, bacterial infection status, and antibiotic adherence, by which all individuals were classified. We denote immune and HAART status by ^superscript^, and bacterial infection status and adherence to antibiotics by _subscript._ (All possible super/sub-script combinations appear in S1 Appendix, which includes details of the mathematical model.) Using tuberculosis (TB) as an example of a pathogen affecting both immunocompromised and immunocompetent individuals, the model follows the progression of bacterial infection throughout an HIV/AIDS-stratified population, including the impact of antibiotic treatment with different levels of adherence (Fig 1). We describe this scenario using a system of ordinary differential equations that appear in S1 Appendix, along with a detailed list of parameters, their condition dependencies, values used, and the reference from which they were estimated.

Considerable variability in antibiotic adherence–and in the prevalence of TB and HIV/AIDS–exists worldwide. The model was therefore run under all combinations of tuberculosis prevalence, host immune status, and antibiotic adherence under parameters representing the HIV/AIDS prevalence in both countries.

In both Indonesia and Swaziland, and under conditions of both high and low TB prevalence (as informed by the World Health Organization’s Global Tuberculosis Report, 2012 [19]), we calculated the projected total number of bacterial infections contracted over 180 days. We then used that calculation to quantify the relative emergence of antibiotic resistance attributable to AIDS-immunocompromised hosts versus those who are HAART-adherent or fully immunocompetent. (Note that we despite the diversity of responses to HAART therapy possible across patients, our models assume a constant efficacy for HAART adherent patients that yields full immunocompetence. However, since our outcomes are reported as relative rates and contributions, the results are robust to the selection of any constant average efficacy for fully adherent HAART treated patients.) This analysis required that we establish a relative probability of emergence corresponding to each immune status/antibiotic adherence category. We accomplished this by multiplying the number of bacterial infections predicted by our model for each subpopulation by: (1) the per-cell per-bacterial generation mutation rate, (2) the expected total number of infected cells per host, (3) the expected number of bacterial generations per infection duration, and (4) the relative success of the mutant strain (see S1 Appendix). [20–23]

To quantify the impact of AIDS-immunocompromised hosts on overall TB prevalence, as well as total emergence, we conducted a trial in which HIV/AIDS prevalence was computationally reduced to zero for each population. We thus calculated the total emergence and total bacterial infections contracted in a fully immunocompetent population without changing the overall host population size or prevalence of infectives. Comparing these two parameters between HIV/AIDS-absent and HIV/AIDS-prevalent populations provided the expected AIDS-attributable increase in emergence. We used this increase to test the neutral assumption that the percentage of emergence associated with each type of infective host should be equivalent to the prevalence of that host category within the total population. For example, in Swaziland, 27.4% of the population is HIV/AIDS+. [6] Within that proportion, we assumed, as an initial condition, that 7% is actively AIDS-immunocompromised. [24] We compared the model projections for the emergence of resistance against the assumption that emergence attributable to actively AIDS-immunocompromised hosts should mirror the prevalence of AIDS-immunocompromised hosts within the population, with the remaining emergent infections originating from those who are fully immunocompetent or HAART-adherent.

Finally, to investigate the full range of AIDS-attributable potential impacts on antibiotic resistance emergence we conducted a trial in which we varied HIV/AIDS prevalence in 5% increments ranging from zero to 30.0%. (At 27.4%, Swaziland’s adult HIV/AIDS prevalence is the currently highest in the world. [6] Based on the nearly 1% increase in prevalence that has occurred in Swaziland since 2013, [6] we expect that 30% of Swaziland’s population could be HIV/AIDS+ within the next few years.) We recognize that considerable variability in TB prevalence can exist between host populations; however, for purposes of illustration, we chose our initial prevalence based on the example of Swaziland’s low infection condition. [19]

## Results

### Relative emergence

**Fig 2:**
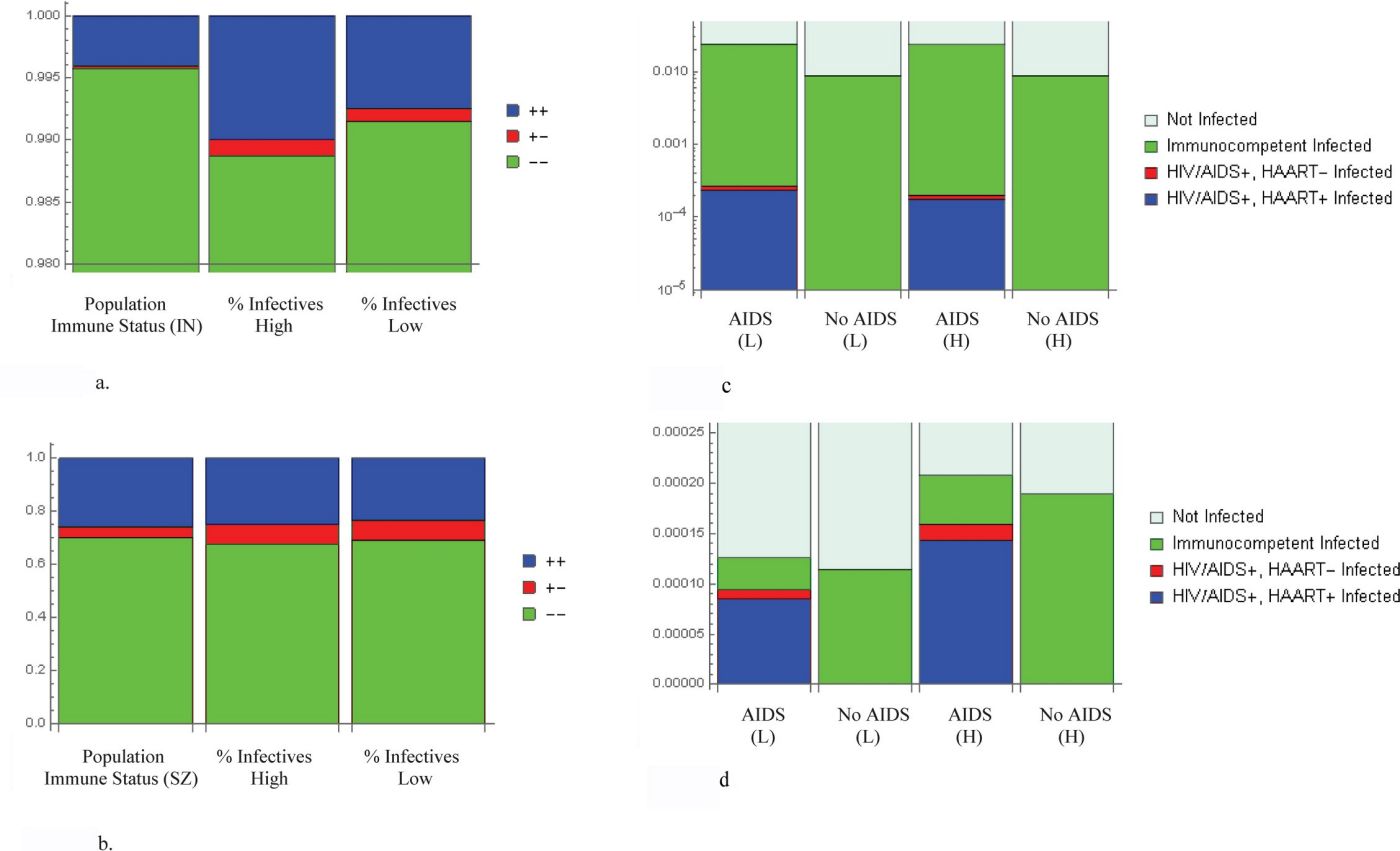
Immune status-based contributions to emergence, and infectivity with and without HIV/AIDS. In both Indonesia (2a) and Swaziland (2b), and under conditions of both high (H) and low (L) TB prevalence, AIDS-immunocompromised hosts are individually responsible for considerably more emergence that would be expected given their prevalence in each country. (We have made the simplifying assumption that HIV/AIDS+, HAART+ hosts are functionally immunocompetent except with respect to loss of immune memory.) Under conditions of high (H) and low (L) TB prevalence, we compare health status-based TB incidence in Indonesia (2c) and Swaziland (2d), visualizing conditions of both actual and zero HIV/AIDS prevalence. In both countries, AIDS increases total incidence. In Swaziland, when TB prevalence is low, we observe an increase in incidence of 9.6%, relative to the AIDS-absent condition; when TB prevalence is high, the relative increase in incidence is 9.9%. In Indonesia, the changes in TB incidence are much more pronounced, with an observed 167.9% increase when TB prevalence is low and a 167.12% increase when TB prevalence is high. Immunocompetent. (We assume 20% adherence to antibiotics, except where specifically noted. [25]).

The relative contribution to total emergence attributable to fully immunocompetent and HAART-adherent hosts, versus those immunocompromised by active AIDS, shows that AIDS-immunocompromised infectives are responsible for 0.09% to 7.52% of emergence, depending on the combination of population-wide HIV/AIDS prevalence and bacterial infection prevalence (detailed in Table 1 and visualized in Fig 2A and 2B).

**Table 1:** AIDS-attributable factor increases in antibiotic-resistant infection.

Population	TB Prevalence	Immune Status	Mean Infective Immune Percentage	Percent Contribution to Emergence	Factor Contribution to Emergence
IN	Low	**--**	99.57%	98.9%	0.99
		**+-**	0.03%	0.14%	5.35
		**++**	0.40%	0.99%	2.49
	High	**--**	99.57%	99.2%	0.99
		**+-**	0.03%	0.09%	3.92
		**++**	0.40%	0.75%	1.86
SZ	Low	**--**	70.28%	67.57%	0.96
		**+-**	3.89%	7.33%	1.82
		**++**	25.81%	25.10%	0.97
	High	**--**	70.28%	69.24%	0.99
		**+-**	3.89%	7.52%	1.93
		**++**	25.81%	23.24%	0.90

Table showing the AIDS-attributable increase in antibiotic-resistant infection in each of the four scenarios (Indonesia or Swaziland, denoted IN or SZ respectively) with low or high tuberculosis (TB) prevalence. The increase due to the population is attributed according to immune and highly-active antiretroviral status: AIDS-negative (--), AIDS-positive HAART-untreated (+-), and AIDS-positive HAART-treated. These data are compiled according to a deterministic mathematical model, and as such there is no standard error to report.

Having separated the infective populations in Indonesia and Swaziland by immune status, we calculated the factor contribution to emergence by dividing the number of emergent infections associated with each class by the mean percentage of individuals appearing in that class over a 180-day period. While the effect is most pronounced in Indonesia, we note that in both countries, under high and low TB conditions, actively AIDS-immunocompromised infectives are responsible for a disproportionately greater number of emergent infections than would be expected based on their prevalence within the population. Despite the conservative assumption of 20% antibiotic adherence, [25] at best, AIDS-immunocompromised infectives are associated with a 1.82-fold increase in emergence.

While these percentages might initially suggest that fully immunocompetent and HAART-adherent hosts pose the greatest health risk with respect to their relative responsibility for emergence, it is important to keep in mind that such hosts represent a much greater portion of the population. In Indonesia, actively AIDS-immunocompromised infectives comprise less than 0.03% of the entire adult population, In contrast, the AIDS-immunocompromised infectives account for less than 4% of all adult Swazilanders. [24] Consequently, a significantly larger proportion of the susceptible population in each country was fully or functionally immunocompetent at the outset of the model.

In analyzing the relative contribution to emergence attributable to each immune status, our results demonstrate that, at the very least, AIDS-immunocompromised infectives are individually responsible for nearly twice as many antibiotic-resistant infections as their comparators (Table 1). This result, which occurs despite our conservative assumption of 20% antibiotic adherence, [25] is troubling in light of the large body of research suggesting that full adherence to dosing instructions is rare in the developing world. [5]

Moreover, the neutral assumption would be that the proportion of drug-resistant TB attributable to AIDS-immunocompromised hosts should be roughly equivalent to the proportion of AIDS-immunocompromised infectives in the population as a whole. Therefore assuming a neutral impact, in Indonesia, we would expect approximately 0.03% of resistant infections to be AIDS-attributable; and, in Swaziland, we would expect approximately 4% of resistant infections to arise in AIDS-immunocompromised hosts. However, this is not the case in either country. As illustrated side-by side (Fig 2A and 2B), with respect to population-wide immune status and mean contribution to total emergence, AIDS-attributable emergence is considerably greater than would be expected based on neutral impact from population AIDS-prevalence alone. In Indonesia, AIDS-immunocompromised hosts are responsible for an average of 0.09% to 0.14% of emergence, depending on bacterial infection prevalence. In Swaziland, AIDS-attributable emergence accounts for an average of 7.32% to 7.52% of total emergence, despite the AIDS-immunocompromised being a smaller portion of the total infective population.

### Knock-out trials

The impact of HIV/AIDS on the emergence of antimicrobial resistance is even clearer when we calculate projected emergence after having reduced HIV/AIDS prevalence to zero. Having used TB data to inform bacterial infection prevalence, and recognizing that HIV-incident TB is common (especially in Swaziland, where an estimated 77% of those with TB are also HIV+ [19]), we expected that computationally reducing HIV/AIDS prevalence to zero would greatly decrease the expected number of new bacterial infections. We find a difference between in total expected infection incidence, as well as the shifts in health-status based infections, when HIV/AIDS+ hosts are absent (Fig 2C and 2D). In both countries, the presence of HIV/AIDS+ hosts results in a disproportionate increase in TB incidence, relative to the percentages of such hosts present in the population. [25] This effect is particularly pronounced in Indonesia where, despite the low initial prevalence of both TB and HIV/AIDS, the continuous availability of susceptibles permits for TB persistence. [17, 19]

### Sensitivity analysis

We performed a sensitivity analysis to determine which parameters had the greatest impact on the disease burden among each of the three populations I^––^, I^+–^, and I^+ +^. In each of the eight possible combinations of Swaziland vs Indonesia, high TB prevalence vs low, and with or without AIDS, we compiled 100 000 samples using Latin hypercube sampling. From these samples, we tested most parameters of the system against the disease burden outcomes to determine which of those parameters were most influential. Note that some of these parameters are basic parameters, from which other parameters are derived. (For example, the number of individuals with AIDS is the total population size, times the HIV-prevalence, times 1–*h*_*h*_. For clarity, we here present only the broad summary of our findings, meaning parameters for which there is a significant positive or negative correlation with disease burden (see S2 Appendix for the full sensitivity analysis, including a table of all parameters and variables). These are listed in Table 2.

**Table 2:** Sensitivity analysis summary.

	*Swaziland*								*Indonesia*							
	Low TB				High TB				Low TB				High TB			
				No AIDS				No AIDS				No Aids				No AIDS
	I ^− −^	I ^+ −^	I ^+ +^	I ^− −^	I ^− −^	I ^+ –^	I ^+ +^	I ^− −^	I ^− −^	I ^+ –^	I ^+ +^	I ^− −^	I ^− −^	I ^+ –^	I ^+ +^	I ^− −^
*c_1_*												X				
*h_a_*	X		O	O	X		O	O		O	O					
*h_h_*		X	O			X	O		X	X				X		
*h_tb_*			O	O		O	O	O		O	O					
*i_tb_*	O				O						X					
*ρ^− −^*												O				
*ρ^+ +^*	O				O				O		O					
$\gamma_{++}^{--}$	O	O	O	O	O	O	O	O					O	O	O	O
$\gamma_{+-}^{--}$	X		X	X	X		X	X			X		X	X	X	X
*θ −_/_*												O				
*θ _/ −_*	X		X	X	X		X	X				X				
*β_0_*	O	O	O	O	O	O	O	O					O	O	O	O

Table summarizing parameters to which disease burden is sensitive. Capital O denotes positive correlation (higher disease burden), while capital X denotes negative correlation. We have noted correlations above 0.1 (in absolute value), except in cases in Indonesia with high TB, where the threshold we use is 0.01. (This is because in a high TB scenario with a very large population, the spread of TB is almost entirely unrestricted–subject to far less influence of the dynamics of the immunocompromised sub-population, resulting in much smaller correlations and the need for a lesser threshold.)

Most of these correlations coincide with *a priori* expectations based on the role of each parameter in the model. The two parameters of which we take particular note are $\gamma_{+-}^{--}$ and θ _*/−*_.

The sensitivity to θ _*/−*_(but not θ –_*/*_) indicates that intervention strategies with partially-adherent individuals may not be effective (and that marginal increases in the rate they drop off into untreated also has no effect), but that strategies targeting untreated individuals may be.

The particular impact of $\gamma_{++}^{--}$ seems to be an artifact of the differential equations model, which assumes that if such individuals are treated with a directly-observed treatment, short course (DOTS) for TB successfully and recover more quickly, they contribute to a greater disease burden–presumably because in the HIV-negative group (the majority of the population) recovers quickly and may become ill again, with no preference to be treated again, potentially ending up in a longer-lasting untreated or partially-adherent state.

### Variation in HIV/AIDS prevalence

**Fig 3:**
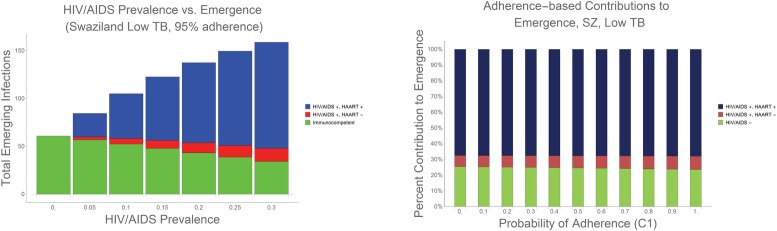
Emergence as a functions of increasing HIV/AIDS prevalence and adherence. 3a. In Using Swaziland’s low TB condition as an example, and assuming 95% antibiotic adherence [25], we found that, as HIV/AIDS prevalence increases from zero to 30%, we observe a corresponding increase in expected population-wide emergence of antibiotic resistance. Our conservative estimate of 20% antibiotic adherence represents a best-case scenario in terms of expected emergence; however, the likelihood of emergence becomes greater as adherence decreases. [26] 3b. Within the developing world, economic, medical and social barriers can limit antibiotic adherence. [26] To measure the impact of antibiotic adherence on emerging resistance within HIV/AIDS prevalent environments, we varied the probability of complete adherence (represented as C_1_ in the model) from 0–100%, while maintaining the original adherence ratios reported in the literature [25, 27] (see S1 Appendix). We use Swaziland’s low TB condition for purposes of illustration, however, we note that the results for Swaziland’s high TB, as well as both the low and high TB conditions in Indonesia, mirror the results presented: antibiotic adherence has very little impact on the emergence of resistance in HIV/AIDS-immunocompromised host populations, relative to the impact of HIV/AIDS prevalence.

When we explored the full range of potential impacts of HIV/AIDS prevalence on emergence of antibiotic resistance, we found that as prevalence increases, we also see a near-linear increase in expected population-wide emergence (Fig 3A). This result, which occurs even if infectives are assumed to adhere to dosing instructions an idealized 95% of the time, demonstrates the importance of the relationship between HIV/AIDS and antibiotic emergence. Even with near-perfect adherence–which is particularly rare within the developing world [26]–emergence of antibiotic resistance increases in response to increased HIV/AIDS prevalence. The relative impact of HIV/AIDS on emergence, versus that of adherence to dosing protocols, is significant (Fig 3B), demonstrating that emergence remains nearly unchanged as antibiotic adherence increases. See S1–S3 Figs for analogous graphs for other model scenarios (Indonesia and/or high TB rates).

## Discussion

Highly AIDS-prevalent populations represent a novel pathogen environment due to the combination of widespread immunoincompetence and antibiotic use. In such populations, antibiotics are used more frequently, and for longer periods of time, to treat and guard against opportunistic infections affecting the AIDS-immunocompromised. [28] This treatment scenario raises the risk of a global public health threat as these populations, following standard antibiotic protocols, may become wellsprings of novel resistant pathogen strains that can propagate into the broader global community. (Note that, while this work is focused specifically upon TB as a particular bacterial pathogen, many bacterial pathogens of accrue antibiotic resistance, and viral and parasitic infections can also be treatment-resistant, presenting additional health concerns to already at-risk populations [29]).

In consideration of these circumstances, as well as the high degree of antibiotic misuse that is characteristic of the developing world, [12] we expected that increased HIV/AIDS prevalence would rapidly accelerate the emergence of antibiotic resistance among circulating bacterial infections. This expectation proved true in both Swaziland and Indonesia where the AIDS-immunocompromised were found to be individually responsible for a 1.82 to 5.35-fold increase in emergent resistant infections than would be expected based on HIV/AIDS prevalence alone.

Despite the considerable difference in HIV/AIDS prevalence that exists between Indonesia and Swaziland, the effect of HIV/AIDS on emergence in Indonesia was still concerning–especially in light of our assumption that only 7% of those with HIV/AIDS are actively immunocompromised due to lack of HAART treatment. [24] Despite this small percentage of AIDS-immunocompromised hosts, we observe at best a nearly two-fold increase over our AIDS prevalence-based expectation of emergence.

Swaziland’s HIV/AIDS prevalence is currently the highest in the world, [6] yet, HIV/AIDS+ Swazilanders still comprise a minority of the population, especially assuming that only 7% of the HIV/AIDS+ population is actively immunocompromised. [24] Despite its size, that portion of the population is responsible for a disproportionate number of emerging drug-resistant infections. This result is particularly troubling when we consider the transmission of resistant strains. For purposes of simplicity, we have modeled emergence as a percentage of total infections only, *i*.*e*., without including parameters for intra-host strain competition or secondary resistant infections arising out of host-to-host or horizontal transmission. We therefore recognize that our results represent a conservative estimate of emergence. Accordingly, future work will address the propagation of resistance that occurs when susceptibles are exposed to resistant infections.

The increased likelihood of resistance emergence that occurs within AIDS-prevalent host populations represents a previously unrecognized global health risk that can be entirely ascribed to the novel environment created by the presence of widespread, population-level immunosuppression. We must begin to consider cross-disease implications for long-term treatments, since emergence of resistance is not solely limited to target bacteria, but also occurs within the greater host microbiome due to both selective pressure and horizontal gene transfer. [30] This type of emergence can result in the replacement of antibiotic-sensitive strains with resistant ones. [31] As exemplified by both viral and bacterial pathogens, the eventual result of these processes is compromised efficacy among previously successful treatment regimens. [31, 32]

## Conclusion

We demonstrated that a significant proportion of antibiotic resistance is attributable to AIDS-immunocompromised hosts. Therefore, we must also consider the associated impact–and potential tradeoff between individual and public health–that arises in the context of antibiotic regulation and policy recommendations for treating infection in the developing world. Even discounting the health behavioral choices made by AIDS-immunocompromised individuals, limited and/or excessive access to antibiotics, coupled with the potential for distribution by medical professionals unfamiliar with optimal administration protocols, [13, 26] ensures the continuing risk of rapid emergence of antibiotic resistance. To foster best medical practices, it is necessary that antibiotic cycling recommendations balance the ethical considerations associated with both personal medicine and public health, such that an active, purposeful, consideration of public health is inherent in antibiotic policy decisions. That said, changes to dosing regulations that may involve the withholding of antibiotics from those unlikely to adhere to prescribing instructions are not without their own set of ethical considerations. The ethical questions surrounding the withholding of potentially life-sustaining treatments–especially among those whose access to antibiotics is already limited by economic constraints–are equally complex, and remain the subject of considerable debate. [33] However, considering the disproportionate increase in drug-resistance that accompanies the presence of HIV/AIDS-affected hosts in an otherwise healthy population, a greater population-level health risk than that imposed by AIDS may occur in the presence of strong selection for antibiotic-resistant microbes.

## Supporting information

S1 Appendix Model description, equations, and parameters used. (DOCX)

S2 Appendix Sensitivity analysis. (DOCX)

S1 Fig Emergence as a functions of increasing HIV/AIDS prevalence and adherence: Analogous to Fig 3, but with high TB rates. (TIF)

S2 Fig Emergence as a functions of increasing HIV/AIDS prevalence and adherence: Analogous to Fig 3, but with respect to Indonesia. (TIF)

S3 Fig Emergence as a functions of increasing HIV/AIDS prevalence and adherence: Analogous to Fig 3, but with high TB rates and with respect to Indonesia. (TIF)

## Abbreviations

AIDS: Autoimmune Deficiency Syndrome
DOTS: directly-observed treatment, short course (TB treatment)
HAART: Highly Active Antiretroviral Treatment
HIV: Human Immunodeficiency Virus
SEIR: Susceptible Exposed Infective Recovered (compartmental model)
TB: Tuberculosis

## References

[1] Levin SA, Andreasen V. Disease transmission dynamics and the evolution of antibiotic resistance in hospitals and communal settings. Proceedings of the National Academy of Sciences. 1999;96(3):800–1. 10.1073/pnas.96.3.800 PMC33521 9927647

[2] Levy SB, Marshall B. Antibacterial resistance worldwide: causes, challenges and responses. Nature Medicine. 2004;10:S122–S9. 10.1038/nm1145 15577930

[3] Kelly CP, LaMont JT. Clostridium difficile—More Difficult Than Ever. New England Journal of Medicine. 2008;359(18):1932–40. 10.1056/NEJMra0707500 18971494

[4] Masur PbH, Kaplan JE, Holmes KK. Guidelines for Preventing Opportunistic Infections among HIV-Infected Persons—2002: Recommendations of the U.S. Public Health Service and the Infectious Diseases Society of America*. Annals of Internal Medicine. 2002;137(2):435–78. 12617574 10.7326/0003-4819-137-5_part_2-200209031-00002

[5] Laxminarayan R, Duse A, Wattal C, Zaidi AKM, Wertheim HFL, Sumpradit N, et al Antibiotic resistance—the need for global solutions. The Lancet Infectious Diseases. 2013;13(12):1057–98. 10.1016/S1473-3099(13)70318-9 24252483

[6] CIA. Central Intelligence Agency World Factbook—Swaziland 2013. Available from: https://www.cia.gov/library/publications/the-world-factbook/geos/wz.html.

[7] Goossens H, Ferech M, Vander Stichele R, Elseviers M. Outpatient antibiotic use in Europe and association with resistance: a cross-national database study. The Lancet. 2005;365(9459):579–87. 10.1016/S0140-6736(05)17907-0 15708101

[8] Dale WB, Peter MH, Workgroup ftSIPGW. Antimicrobial Prophylaxis for Surgery: An Advisory Statement from the National Surgical Infection Prevention Project. Clinical Infectious Diseases. 2004;38(12):1706–15. 10.1086/421095 15227616

[9] Willing BP, Russell SL, Finlay BB. Shifting the balance: antibiotic effects on host–microbiota mutualism. Nature Reviews Microbiology. 2011;9(4):233–43. 10.1038/nrmicro2536 21358670

[10] Maiden MC. Horizontal genetic exchange, evolution, and spread of antibiotic resistance in bacteria. Clinical Infectious Diseases. 1998;27(Supplement 1):S12–S20. 9710667 10.1086/514917

[11] Laxminarayan R, Weitzman ML. On the implications of endogenous resistance to medications. Journal of Health Economics. 2002;21(4):709–18. 12146599 10.1016/s0167-6296(02)00034-6

[12] Morgan DJ, Okeke IN, Laxminarayan R, Perencevich EN, Weisenberg S. Non-prescription antimicrobial use worldwide: a systematic review. The Lancet Infectious Diseases. 2011;11(9):692–701. 10.1016/S1473-3099(11)70054-8 21659004 PMC3543997

[13] Okeke IN, Lamikanra A, Edelman R. Socioeconomic and behavioral factors leading to acquired bacterial resistance to antibiotics in developing countries. Emerging Infectious Diseases. 1999;5(1):18 10.3201/eid0501.990103 10081668 PMC2627681

[14] Connor BA, Keystone JS. Antibiotic Self-treatment of Travelers' Diarrhea: Helpful or Harmful? Clinical Infectious Diseases. 2015. 10.1093/cid/ciu961 25613288

[15] Galvani AP. Epidemiology meets evolutionary ecology. Trends in Ecology & Evolution. 2003;18(3):132–9.

[16] McKendrick AG. Applications of Mathematics to Medical Problems. Proceedings of the Edinburgh Mathematical Society. 1925;44:98–130.

[17] CIA. Central Intelligence Agency World Factbook—Indonesia 2013 Available from: https://www.cia.gov/library/publications/the-world-factbook/geos/id.html.

[18] Coker RJ, Hunter BM, Rudge JW, Liverani M, Hanvoravongchai P. Emerging infectious diseases in southeast Asia: regional challenges to control. The Lancet. 2011;377(9765):599–609. 10.1016/S0140-6736(10)62004-1 PMC7159088 21269678

[19] Organization WH. Global tuberculosis report 2012. Geneva: World Health Organization; 2012. Available from: h ttp://apps who int/iris/bitstream/10665/75938/1/9789241564502_eng pdf(Accessed 2013 June 6). 2012:66.

[20] Billington O, McHugh T, Gillespie S. Physiological cost of rifampin resistance induced in vitro in Mycobacterium tuberculosis. Antimicrobial Agents and Chemotherapy. 1999;43(8):1866–9. 10428904 10.1128/aac.43.8.1866 PMC89382

[21] Stone KC, Mercer RR, Gehr P, Stockstill B, Crapo JD. Allometric Relationships of Cell Numbers and Size in the Mammalian Lung. American Journal of Respiratory Cell and Molecular Biology. 1992;6(2):235–43. 10.1165/ajrcmb/6.2.235 1540387

[22] Dormans J, Burger M, Aguilar D, Hernandez-Pando R, Kremer K, Roholl P, et al Correlation of virulence, lung pathology, bacterial load and delayed type hypersensitivity responses after infection with different Mycobacterium tuberculosis genotypes in a BALB/c mouse model. Clinical & Experimental Immunology. 2004;137(3):460–8. 15320894 10.1111/j.1365-2249.2004.02551.x PMC1809137

[23] Gill WP, Harik NS, Whiddon MR, Liao RP, Mittler JE, Sherman DR. A replication clock for Mycobacterium tuberculosis. Nat Med. 2009;15(2):211–4. 10.1038/nm.1915 19182798 PMC2779834

[24] Morgan D, Mahe C, Mayanja B, Okongo JM, Lubega R, Whitworth JA. HIV-1 infection in rural Africa: is there a difference in median time to AIDS and survival compared with that in industrialized countries? AIDS. 2002;16(4):597–603. 11873003 10.1097/00002030-200203080-00011

[25] Blower SM, Chou T. Modeling the emergence of the'hot zones': tuberculosis and the amplification dynamics of drug resistance. Nature Medicine. 2004;10(10):1111–6. 10.1038/nm1102 15378053

[26] Laxminarayan R, Heymann DL. Challenges of drug resistance in the developing world2012 2012-4-03 10:44:01. 10.1136/bmj.e1567 22491075

[27] Trostle J. Inappropriate distribution of medicines by professionals in developing countries. Social Science & Medicine. 1996;42(8):1117–20. 8737428 10.1016/0277-9536(95)00384-3

[28] Meyer CN, Skinhoj P, Prag J. Bacteremia in HIV-Positive and AIDS Patients—Incidence, Species DIstribution, Risk-Factors, Outcome, and Influence of Long-Term Prophylactic Antibiotic-Treatment. Scand J Infect Dis. 1994;26(6):635–42. 7747085 10.3109/00365549409008630

[29] Sullivan JC, De Meyer S, Bartels DJ, Dierynck I, Zhang EZ, Spanks J, et al Evolution of Treatment-Emergent Resistant Variants in Telaprevir Phase 3 Clinical Trials. Clinical Infectious Diseases. 2013;57(2):221–9. 10.1093/cid/cit226 23575197

[30] Martínez JL, Baquero F. Interactions among strategies associated with bacterial infection: pathogenicity, epidemicity, and antibiotic resistance. Clinical Microbiology Reviews. 2002;15(4):647–79. 10.1128/CMR.15.4.647-679.2002 12364374 PMC126860

[31] Lipsitch M, Cohen T, Murray M, Levin BR. Antiviral resistance and the control of pandemic influenza. PLoS medicine. 2007;4(1):e15 10.1371/journal.pmed.0040015 17253900 PMC1779817

[32] Moran GJ, Krishnadasan A, Gorwitz RJ, Fosheim GE, McDougal LK, Carey RB, et al Methicillin-Resistant S. aureus Infections among Patients in the Emergency Department. New England Journal of Medicine. 2006;355(7):666–74. 10.1056/NEJMoa055356 16914702

[33] Selgelid MJ. Ethics and Drug Resistance. Bioethics. 2007;21(4):218–29. 10.1111/j.1467-8519.2006.00542.x 17845480

