# Supplementary material for: Emergence of antibiotic resistance in immunocompromised host populations: A case study of emerging antibiotic resistant tuberculosis in AIDS patients
Source: PLoS One. 2019 Feb 28;14(2):e0212969. doi: 10.1371/journal.pone.0212969 (PMC6394933; doi:10.1371/journal.pone.0212969)
Supplement: S2 Appendix — (DOCX) [file pone.0212969.s002.docx]

**S2 Appendix. Sensitivity Analysis**

We performed a sensitivity analysis to determine which parameters had the greatest impact on the disease burden among each of the three populations I^– –^, I^+ –^, and I^+ +^. In each of the eight possible combinations of Swaziland vs. Indonesia, high TB prevalence vs. low, and with or without AIDS, we compiled 100,000 samples using Latin hypercube sampling. From these samples, we tested most parameters of the system against the disease burden outcomes to determine which of those parameters were most influential. Note that some of these parameters are basic parameters, from which other parameters are derived. (For example, the number of individuals with AIDS is the total population size, times the HIV-prevalence, times 1–*h_h_*. A table with the full sensitivity analysis results can be found in Appendix X, however for clarity, we here present only the broad summary of our findings (i.e. positive or negative correlation between disease burden and parameter value).

|  | Swaziland | | | | | | | | Indonesia | | | | | | | |
| --- | --- | --- | --- | --- | --- | --- | --- | --- | --- | --- | --- | --- | --- | --- | --- | --- |
|  | Low TB | | | | High TB | | | | Low TB | | | | High TB | | | |
|  |  |  |  | No AIDS |  |  |  | No AIDS |  |  |  | No Aids |  |  |  | No AIDS |
|  | I ^– –^ | I ^+ –^ | I ^+ +^ | I ^– –^ | I ^– –^ | I ^+ –^ | I ^+ +^ | I ^– –^ | I ^– –^ | I ^+ –^ | I ^+ +^ | I ^– –^ | I ^– –^ | I ^+ –^ | I ^+ +^ | I ^– –^ |
| c_1_ |  |  |  |  |  |  |  |  |  |  |  | X |  |  |  |  |
| h_a_ | X |  | O | O | X |  | O | O |  | O | O |  |  |  |  |  |
| h_h_ |  | X | O |  |  | X | O |  | X | X |  |  |  | X |  |  |
| h_tb_ |  |  | O | O |  | O | O | O |  | O | O |  |  |  |  |  |
| i_tb_ | O |  |  |  | O |  |  |  |  |  | X |  |  |  |  |  |
| ρ^– –^ |  |  |  |  |  |  |  |  |  |  |  | O |  |  |  |  |
| ρ^++^ | O |  |  |  | O |  |  |  | O |  | O |  |  |  |  |  |
| $\gamma_{++}^{--}$ | O | O | O | O | O | O | O | O |  |  |  |  | O | O | O | O |
| $\gamma_{+-}^{--}$ | X |  | X | X | X |  | X | X |  |  | X |  | X | X | X | X |
| θ _– /_ |  |  |  |  |  |  |  |  |  |  |  | O |  |  |  |  |
| θ _/ –_ | X |  | X | X | X |  | X | X |  |  |  | X |  |  |  |  |
| β_0_ | O | O | O | O | O | O | O | O |  |  |  |  | O | O | O | O |

Table X: Sensitivity Analysis Summary. Capital O denotes positive correlation (higher disease burden), while capital X denotes negative correlation. We have noted correlations above 0.1 (in absolute value), except in cases in Indonesia with high TB, where the threshold we use is 0.01. (This is because in a high TB scenario with a very large population, the spread of TB is almost entirely unrestricted – subject to far less influence of the dynamics of this system, such that no correlation is above 0.1.)

Most of these correlations coincide with *a priori* expectations based on the role of each parameter in the model. The two parameters of which we take particular note are $\gamma_{+-}^{--}$ and θ *_/ –_ .*

The sensitivity to θ *_/ –_*  (but not θ *_– /_* ) indicates that intervention strategies with partially-adherent individuals may not be effective (and that marginal increases in the rate they drop off into untreated also has no effect), but that strategies targeting untreated individuals may be.

The particular impact of $\gamma_{++}^{--}$ seems to be an artifact of the ODE model, which assumes that if such individuals are treated with DOTS successfully and recover more quickly, they contribute to a greater disease burden – presumably because in the HIV-negative group (the majority of the population) recovers quickly and may become ill again, with no preference to be treated again, potentially ending up in a longer-lasting untreated or partially-adherent state.

**Appendix X: Sensitivity Analysis**

Included below are tables indicating the correlation between the system’s parameters and the disease burden among each group of immune status. Columns are given for the raw correlation and for the normalized correlation (relative correlation, with all correlations weighted out of a sum total of 1). In cases with no AIDS, columns for those groups are not included.

The parameters we examine are as follows:

- *c*_1_ : probability of early TB detection and complete DOTS treatment
- *h*_a_ : AIDS prevalence
- *h*_h_ : portion of HIV-positive individuals without AIDS
- *h*_tb_ : TB prevalence among AIDS-positive individuals
- *d*_tb_ : TB-attributable death-rate
- *i*_tb_ : TB prevalence
- ρ*^– –^* , ρ*^+ –^* , ρ^++^ : rate of immune memory loss in each immunocompetence category
- $\gamma_{++}^{--}$ , $\gamma_{+0}^{--}$ , $\gamma_{+-}^{--}$ , $\gamma_{++}^{+-}$ , $\gamma_{+0}^{+-}$ , $\gamma_{+-}^{+-}$ : TB recovery rate in each immunocompetence / antibacterial treatment category
- *θ* *_– /_* , *θ* *_/ –_* : transition rate between partial antibiotic adherence and no untreated categories
- *β*_0_ : base TB transmission rate (all other rates computed as a multiple of this)
- *ζ*_0_ : base rate of transition from exposed to infective (other rates a multiple of this)
- *ω* : ambient mortality rate
- *ω*_b_ : mortality rate due to TB
- *ω*_v_ : mortality rate due to AIDS
- α : birth rate

In the tables below, we have noted all correlations above 0.1, except in cases in Indonesia with high TB, where the threshold is 0.01. (This is because in a high TB scenario with a very large population, the spread of TB is almost entirely unrestricted – subject to far less influence of the dynamics of the immunocompromised sub-population, so much that no correlation is above 0.1.)

Swaziland, low TB:

|  | I ^– –^ | I ^– –^ (norm) | I ^+ –^ | I ^+ –^ (norm) | I ^+ +^ | I ^+ +^ (norm) |
| --- | --- | --- | --- | --- | --- | --- |
| c_1_ | -0.0424825825 | 0.013631499 | 0.0306896806 | 0.0148983952 | -0.0699388544 | 0.0184911256 |
| h_a_ | **-0.3166644267** | **0.1016089554** | 0.073822376 | 0.0358372884 | **0.2243146264** | **0.0593065181** |
| h_h_ | -0.0242293671 | 0.0077745414 | **-0.9806543637** | **0.4760615299** | **0.3811831228** | **0.1007809617** |
| h_tb_ | 0.0376786094 | 0.0120900354 | 0.0997037222 | 0.0484014637 | **0.3807868929** | **0.1006762026** |
| d_tb_ | -0.0287343215 | 0.0092200581 | 0.030283695 | 0.0147013084 | -0.0596970809 | 0.0157833043 |
| i_tb_ | **0.3305757292** | **0.106072712** | 0.0211152456 | 0.0102504577 | -0.0530383774 | 0.0140228105 |
| ρ^– –^ | -0.0062305179 | 0.0019992028 | 0.027653324 | 0.013424387 | -0.038979502 | 0.010305786 |
| ρ^+ –^ | -0.025511516 | 0.0081859479 | 0.0268993031 | 0.0130583454 | -0.056515143 | 0.0149420321 |
| ρ^++^ | **0.1134523893** | **0.0364037694** | 0.0474651562 | 0.0230420989 | 0.0862788518 | 0.0228112556 |
| $\gamma_{++}^{--}$ | **0.4689948711** | **0.1504876296** | **0.111607422** | **0.0541801495** | **0.3782684238** | **0.1000103448** |
| $\gamma_{+0}^{--}$ | -0.0688872515 | 0.0221040353 | 0.0316788216 | 0.0153785766 | -0.0810431091 | 0.0214269782 |
| $\gamma_{+-}^{--}$ | **-0.547102441** | **0.175550213** | -0.0486546001 | 0.023619518 | **-0.5121869104** | **0.135417038** |
| $\gamma_{++}^{+-}$ | -0.0271391459 | 0.0087082098 | 0.0272399392 | 0.0132237082 | -0.0583163631 | 0.0154182565 |
| $\gamma_{+0}^{+-}$ | -0.0280963701 | 0.0090153569 | 0.0278235354 | 0.0135070167 | -0.060204201 | 0.0159173817 |
| $\gamma_{+-}^{+-}$ | -0.0262984653 | 0.0084384584 | 0.0279260216 | 0.0135567689 | -0.0584528516 | 0.0154543427 |
| θ _– /_ | 0.0586549115 | 0.0188207572 | 0.0374299426 | 0.0181704751 | 0.0392809751 | 0.0103854925 |
| θ _/ –_ | **-0.1273940889** | **0.0408772796** | 0.0138497937 | 0.0067234229 | **-0.1669670963** | **0.0441444113** |
| β_0_ | **0.4966699514** | **0.1593678061** | **0.1049134713** | **0.0509305516** | **0.4022148117** | **0.1063415275** |
| ζ_0_ | 0.0531799148 | 0.0170639805 | 0.0265973525 | 0.0129117626 | -0.0545252678 | 0.0144159292 |
| ω | -0.02901521 | 0.0093101875 | 0.0235373042 | 0.0114262532 | -0.0600719014 | 0.0158824031 |
| ω_b_ | -0.0524852085 | 0.0168410682 | 0.0058495275 | 0.0028396702 | -0.0824133766 | 0.0217892631 |
| ω_v_ | -0.025520853 | 0.0081889439 | 0.0306955681 | 0.0149012533 | -0.0633093457 | 0.0167383506 |
| α | -0.0223288648 | 0.0071647221 | 0.0332124998 | 0.0161231052 | -0.0594122642 | 0.0157080016 |

Swaziland, high TB:

|  | I ^– –^ | I ^– –^ (norm) | I ^+ –^ | I ^+ –^ (norm) | I ^+ +^ | I ^+ +^ (norm) |
| --- | --- | --- | --- | --- | --- | --- |
| c_1_ | -0.0504148984 | 0.0162616748 | 0.0315039104 | 0.0152950326 | -0.0746302182 | 0.0197491363 |
| h_a_ | **-0.3099639422** | **0.0999810171** | 0.0789332252 | 0.0383217905 | **0.2299984655** | **0.0608636979** |
| h_h_ | -0.0209115341 | 0.0067451602 | **-0.9809644577** | **0.4762546355** | **0.3818655839** | **0.1010517678** |
| h_tb_ | 0.0403975769 | 0.0130305183 | **0.1054353913** | **0.0511884946** | **0.3786504044** | **0.100200946** |
| d_tb_ | -0.0244716623 | 0.0078935042 | 0.0263068241 | 0.0127718663 | -0.0581434363 | 0.0153862963 |
| i_tb_ | **0.3163715787** | **0.1020478447** | 0.0245014074 | 0.0118953432 | -0.06531965 | 0.0172853129 |
| ρ^– –^ | 0.0041790387 | 0.0013479779 | 0.0298012263 | 0.0144683857 | -0.0314087886 | 0.008311599 |
| ρ^+ –^ | -0.0269658946 | 0.0086980361 | 0.0297369557 | 0.0144371826 | -0.0578055762 | 0.0152968895 |
| ρ^++^ | **0.1125710689** | **0.0363105782** | 0.0474262324 | 0.0230252614 | 0.0821096671 | 0.021728397 |
| $\gamma_{++}^{--}$ | **0.4696801416** | **0.1514985838** | **0.1001419061** | **0.048618527** | **0.3831681485** | **0.1013964609** |
| $\gamma_{+0}^{--}$ | -0.0628680412 | 0.0202785223 | 0.0314743577 | 0.0152806849 | -0.0780856436 | 0.0206635336 |
| $\gamma_{+-}^{--}$ | **-0.5493733363** | **0.1772041759** | -0.0491365507 | 0.0238556146 | **-0.512419128** | **0.1355997003** |
| $\gamma_{++}^{+-}$ | -0.0244570762 | 0.0078887994 | 0.0252396738 | 0.0122537688 | -0.0598960488 | 0.0158500841 |
| $\gamma_{+0}^{+-}$ | -0.0258176735 | 0.0083276695 | 0.0225377504 | 0.0109419949 | -0.0577271103 | 0.0152761254 |
| $\gamma_{+-}^{+-}$ | -0.0259758129 | 0.0083786784 | 0.0249478017 | 0.0121120659 | -0.0578974548 | 0.0153212031 |
| θ _– /_ | 0.0582215311 | 0.0187797582 | 0.0416077212 | 0.0202003956 | 0.0346178251 | 0.0091607952 |
| θ _/ –_ | **-0.1285568021** | **0.0414668872** | 0.0129032287 | 0.0062644701 | **-0.1688928561** | **0.044693532** |
| β_0_ | **0.4927342488** | **0.1589348458** | **0.1037629621** | **0.0503765364** | **0.3996418036** | **0.1057558273** |
| ζ_0_ | 0.0560179195 | 0.0180689681 | 0.0271831957 | 0.0131973415 | -0.0515411595 | 0.0136391587 |
| ω | -0.0246766935 | 0.0079596385 | 0.0282212819 | 0.0137013286 | -0.0596649242 | 0.0157889224 |
| ω_b_ | -0.0521218197 | 0.0168122541 | 0.0070227078 | 0.0034094988 | -0.0843388568 | 0.0223182998 |
| ω_v_ | -0.0317382273 | 0.0102373851 | 0.0263724926 | 0.0128037482 | -0.062608708 | 0.0165679257 |
| α | -0.0287664984 | 0.0092788334 | 0.0301710932 | 0.0146479548 | -0.0591867569 | 0.0156623866 |

Indonesia, low TB:

|  | I ^– –^ | I ^– –^ (norm) | I ^+ –^ | I ^+ –^ (norm) | I ^+ +^ | I ^+ +^ (norm) |
| --- | --- | --- | --- | --- | --- | --- |
| c_1_ | -0.0024427121 | 0.0018041418 | 0.0256202614 | 0.0128759503 | -0.059446417 | 0.0262839386 |
| h_a_ | -0.0032888131 | 0.0024290563 | **0.1061580678** | **0.0533517587** | **0.4567258801** | **0.2019390839** |
| h_h_ | **-0.6115916198** | **0.4517102203** | **-0.9896667215** | **0.4973758583** | 0.0060879521 | 0.0026917579 |
| h_tb_ | 0.0140139536 | 0.0103504461 | **0.1022716702** | **0.0513985755** | **0.3940603865** | **0.1742318466** |
| d_tb_ | 0.0209004919 | 0.0154367155 | 0.03254133 | 0.0163542651 | -0.0261144104 | 0.0115463571 |
| i_tb_ | 0.0212894663 | 0.0157240047 | -0.0068877908 | 0.0034615904 | **-0.2875633951** | **0.1271447297** |
| ρ^– –^ | 0.0357280245 | 0.0263880559 | 0.0342628692 | 0.0172194574 | -0.0177225409 | 0.0078359336 |
| ρ^+ –^ | 0.0194548819 | 0.0143690147 | 0.0305784701 | 0.0153677925 | -0.0260374932 | 0.0115123485 |
| ρ^++^ | **0.2100599203** | **0.1551463588** | 0.048863067 | 0.0245570649 | **0.1385046976** | **0.0612391655** |
| $\gamma_{++}^{--}$ | 0.0595817143 | 0.0440059485 | 0.0487880989 | 0.0245193882 | 0.085984647 | 0.0380176855 |
| $\gamma_{+0}^{--}$ | 0.0156656554 | 0.0115703623 | 0.030588003 | 0.0153725834 | -0.0264141142 | 0.0116788697 |
| $\gamma_{+-}^{--}$ | -0.0253412288 | 0.0187165613 | 0.0122200298 | 0.0061414087 | **-0.1384147164** | **0.0611993807** |
| $\gamma_{++}^{+-}$ | -0.0336900764 | 0.0248828652 | 0.0261587635 | 0.0131465848 | -0.0665551125 | 0.0294270131 |
| $\gamma_{+0}^{+-}$ | 0.0150426403 | 0.011110215 | 0.0314872184 | 0.0158245012 | -0.0305639453 | 0.0135136969 |
| $\gamma_{+-}^{+-}$ | 0.0604107814 | 0.044618282 | 0.0278678407 | 0.0140055141 | 0.0090362708 | 0.0039953424 |
| θ _– /_ | 0.0304057904 | 0.0224571525 | 0.0345168204 | 0.0173470854 | -0.0152862005 | 0.0067587178 |
| θ _/ –_ | -0.0019247268 | 0.0014215675 | 0.0316534606 | 0.0159080494 | -0.0398143202 | 0.0176037043 |
| β_0_ | 0.0015676392 | 0.0011578292 | 0.0431614196 | 0.0216915934 | 0.0416117161 | 0.018398414 |
| ζ_0_ | 0.0070803897 | 0.0052294444 | 0.0164723979 | 0.0082785173 | -0.1130057433 | 0.0499649292 |
| ω | 0.0189302921 | 0.0139815624 | 0.0316283943 | 0.0158954519 | -0.02432401 | 0.0107547404 |
| ω_b_ | 0.011561412 | 0.0085390443 | 0.031421654 | 0.0157915506 | -0.0354429438 | 0.0156709219 |
| ω_v_ | 0.0180587413 | 0.0133378512 | 0.0287486925 | 0.0144482029 | -0.0267350675 | 0.0118207776 |
| α | 0.0227524831 | 0.0168045618 | 0.0321789054 | 0.0161721217 | -0.0220322729 | 0.0097414602 |

Indonesia, high TB:

|  | I ^– –^ | I ^– –^ (norm) | I ^+ –^ | I ^+ –^ (norm) | I ^+ +^ | I ^+ +^ (norm) |
| --- | --- | --- | --- | --- | --- | --- |
| c_1_ | -0.0048815996 | 0.020925229 | -0.0007389004 | 0.0028612887 | -0.0051632072 | 0.0211529557 |
| h_a_ | -0.000823661 | 0.0035306656 | 0.0079381388 | 0.0307393348 | 0.0055977716 | 0.0229333066 |
| h_h_ | -0.0077809749 | 0.0333535511 | **-0.0670752092** | **0.2597393892** | 0.0078262864 | 0.0320632278 |
| h_tb_ | -0.0024832884 | 0.0106447441 | 0.0064827465 | 0.0251035313 | 0.0032548709 | 0.0133347619 |
| d_tb_ | -0.0047582426 | 0.0203964531 | 0.0036747902 | 0.0142301125 | -0.0064620573 | 0.0264741672 |
| i_tb_ | 0.0096546906 | 0.0413853303 | 0.0014708226 | 0.0056955552 | 0.0031563442 | 0.0129311118 |
| ρ^– –^ | 0.0026894329 | 0.0115283934 | 0.0059169398 | 0.0229125237 | 0.0018629824 | 0.0076323846 |
| ρ^+ –^ | -0.0016288984 | 0.0069823574 | -0.000468401 | 0.0018138176 | -0.0020026271 | 0.0082044902 |
| ρ^++^ | -0.0040289997 | 0.0172705156 | -0.0002135996 | 0.0008271346 | -0.0079256217 | 0.032470191 |
| $\gamma_{++}^{--}$ | **0.0484435668** | **0.2076558535** | **0.0398145766** | **0.1541763931** | **0.047382539** | **0.1941197987** |
| $\gamma_{+0}^{--}$ | -0.0028222336 | 0.0120976501 | -0.0043924575 | 0.0170091789 | -0.0035209456 | 0.0144248337 |
| $\gamma_{+-}^{--}$ | **-0.0565355552** | **0.2423425802** | **-0.0420136813** | **0.162692119** | **-0.0565531684** | **0.2316906163** |
| $\gamma_{++}^{+-}$ | 0.0001474962 | 0.0006322503 | 0.0048477661 | 0.0187722978 | 0.0005129876 | 0.00210164 |
| $\gamma_{+0}^{+-}$ | -0.0007158063 | 0.0030683408 | -0.0006628285 | 0.0025667109 | -0.0010543318 | 0.0043194534 |
| $\gamma_{+-}^{+-}$ | 0.0009466804 | 0.0040579946 | 0.0028544012 | 0.0110532705 | -0.0002058744 | 0.0008434393 |
| θ _– /_ | -0.0021653896 | 0.0092820546 | 0.0015627693 | 0.0060516062 | -0.0037756741 | 0.0154684216 |
| θ _/ –_ | -0.0079929867 | 0.0342623506 | -0.0041444736 | 0.0160488959 | -0.0087977456 | 0.0360431636 |
| β_0_ | **0.0508488656** | **0.2179662913** | **0.0398972632** | **0.1544965854** | **0.0507985572** | **0.208114759** |
| ζ_0_ | 0.0001032421 | 0.0004425524 | 0.0006485166 | 0.0025112901 | -0.0009250958 | 0.0037899915 |
| ω | -0.0008113475 | 0.0034778828 | 0.0055425905 | 0.0214629084 | -0.0020076978 | 0.0082252639 |
| ω_b_ | -0.0012673699 | 0.0054326467 | -0.0022702273 | 0.0087911384 | -0.0017394616 | 0.0071263371 |
| ω_v_ | 0.0021114602 | 0.0090508834 | 0.0015421855 | 0.0059718984 | 0.0010670397 | 0.004371516 |
| α | 0.0006957681 | 0.002982446 | 0.003120272 | 0.0120828178 | 0.0005583594 | 0.0022875224 |

Swaziland, low TB, no AIDS:

|  | I ^– –^ | I ^– –^ (norm) | I ^+ –^ | I ^+ –^ (norm) | I ^+ +^ | I ^+ +^ (norm) |
| --- | --- | --- | --- | --- | --- | --- |
| c_1_ | -0.0575031875 | 0.0183186335 | n/a | n/a | n/a | n/a |
| h_a_ | **0.1001184932** | **0.0318944751** | n/a | n/a | n/a | n/a |
| h_h_ | -0.043108746 | 0.0137330356 | n/a | n/a | n/a | n/a |
| h_tb_ | **0.3375074016** | **0.1075188118** | n/a | n/a | n/a | n/a |
| d_tb_ | -0.038227194 | 0.0121779329 | n/a | n/a | n/a | n/a |
| i_tb_ | 0.0759822357 | 0.0242054535 | n/a | n/a | n/a | n/a |
| ρ^– –^ | -0.0019005943 | 0.0006054671 | n/a | n/a | n/a | n/a |
| ρ^+ –^ | -0.0398019784 | 0.0126796076 | n/a | n/a | n/a | n/a |
| ρ^++^ | -0.0421692691 | 0.013433749 | n/a | n/a | n/a | n/a |
| $\gamma_{++}^{--}$ | **0.4756390984** | **0.1515230494** | n/a | n/a | n/a | n/a |
| $\gamma_{+0}^{--}$ | -0.0851753367 | 0.0271340745 | n/a | n/a | n/a | n/a |
| $\gamma_{+-}^{--}$ | **-0.5850749362** | **0.1863857255** | n/a | n/a | n/a | n/a |
| $\gamma_{++}^{+-}$ | -0.0385234712 | 0.0122723171 | n/a | n/a | n/a | n/a |
| $\gamma_{+0}^{+-}$ | -0.0333605135 | 0.0106275677 | n/a | n/a | n/a | n/a |
| $\gamma_{+-}^{+-}$ | -0.0371732445 | 0.011842179 | n/a | n/a | n/a | n/a |
| θ _– /_ | 0.0665629098 | 0.0212047645 | n/a | n/a | n/a | n/a |
| θ _/ –_ | **-0.1627767933** | **0.0518553588** | n/a | n/a | n/a | n/a |
| β_0_ | **0.5133986412** | **0.1635520039** | n/a | n/a | n/a | n/a |
| ζ_0_ | -0.0054519851 | 0.001736824 | n/a | n/a | n/a | n/a |
| ω | -0.0406417994 | 0.0129471471 | n/a | n/a | n/a | n/a |
| ω_b_ | -0.0681947786 | 0.0217246245 | n/a | n/a | n/a | n/a |
| ω_v_ | -0.0356618036 | 0.0113606834 | n/a | n/a | n/a | n/a |
| α | -0.0317825761 | 0.0101248885 | n/a | n/a | n/a | n/a |

Swaziland, high TB, no AIDS:

|  | I ^– –^ | I ^– –^ (norm) | I ^+ –^ | I ^+ –^ (norm) | I ^+ +^ | I ^+ +^ (norm) |
| --- | --- | --- | --- | --- | --- | --- |
| c_1_ | -0.0641197854 | 0.0203219667 | n/a | n/a | n/a | n/a |
| h_a_ | **0.112681793** | **0.0357130896** | n/a | n/a | n/a | n/a |
| h_h_ | -0.0421414148 | 0.0133561961 | n/a | n/a | n/a | n/a |
| h_tb_ | **0.3529960873** | **0.1118777095** | n/a | n/a | n/a | n/a |
| d_tb_ | -0.0352417901 | 0.011169446 | n/a | n/a | n/a | n/a |
| i_tb_ | 0.0638514962 | 0.0202369358 | n/a | n/a | n/a | n/a |
| ρ^– –^ | -0.0088036173 | 0.0027901968 | n/a | n/a | n/a | n/a |
| ρ^+ –^ | -0.0347019986 | 0.0109983659 | n/a | n/a | n/a | n/a |
| ρ^++^ | -0.0390747818 | 0.012384265 | n/a | n/a | n/a | n/a |
| $\gamma_{++}^{--}$ | **0.4744422941** | **0.1503685707** | n/a | n/a | n/a | n/a |
| $\gamma_{+0}^{--}$ | -0.0855795473 | 0.0271233707 | n/a | n/a | n/a | n/a |
| $\gamma_{+-}^{--}$ | **-0.5807106838** | **0.1840490121** | n/a | n/a | n/a | n/a |
| $\gamma_{++}^{+-}$ | -0.0350167645 | 0.011098127 | n/a | n/a | n/a | n/a |
| $\gamma_{+0}^{+-}$ | -0.0434623595 | 0.0137748531 | n/a | n/a | n/a | n/a |
| $\gamma_{+-}^{+-}$ | -0.0321141069 | 0.0101781658 | n/a | n/a | n/a | n/a |
| θ _– /_ | 0.0687671804 | 0.0217949006 | n/a | n/a | n/a | n/a |
| θ _/ –_ | **-0.1557677485** | **0.0493686461** | n/a | n/a | n/a | n/a |
| β_0_ | **0.5067322135** | **0.1606024582** | n/a | n/a | n/a | n/a |
| ζ_0_ | -0.0069274909 | 0.0021955819 | n/a | n/a | n/a | n/a |
| ω | -0.0399803374 | 0.0126712695 | n/a | n/a | n/a | n/a |
| ω_b_ | -0.0656687453 | 0.0208128902 | n/a | n/a | n/a | n/a |
| ω_v_ | -0.0386365202 | 0.0122453634 | n/a | n/a | n/a | n/a |
| α | -0.0418899237 | 0.0132764891 | n/a | n/a | n/a | n/a |

Indonesia, low TB, no AIDS:

|  | I ^– –^ | I ^– –^ (norm) | I ^+ –^ | I ^+ –^ (norm) | I ^+ +^ | I ^+ +^ (norm) |
| --- | --- | --- | --- | --- | --- | --- |
| c_1_ | **-0.4317742001** | **0.1960210807** | n/a | n/a | n/a | n/a |
| h_a_ | -0.0180266217 | 0.0081839023 | n/a | n/a | n/a | n/a |
| h_h_ | -0.0232647816 | 0.0105619734 | n/a | n/a | n/a | n/a |
| h_tb_ | -0.0156422584 | 0.0071014257 | n/a | n/a | n/a | n/a |
| d_tb_ | -0.0201803912 | 0.0091616917 | n/a | n/a | n/a | n/a |
| i_tb_ | -0.023628314 | 0.0107270135 | n/a | n/a | n/a | n/a |
| ρ^– –^ | **0.8693965458** | **0.3946971599** | n/a | n/a | n/a | n/a |
| ρ^+ –^ | -0.0134628286 | 0.0061119868 | n/a | n/a | n/a | n/a |
| ρ^++^ | -0.0183459262 | 0.0083288633 | n/a | n/a | n/a | n/a |
| $\gamma_{++}^{--}$ | 0.0072240135 | 0.0032796284 | n/a | n/a | n/a | n/a |
| $\gamma_{+0}^{--}$ | -0.0311817402 | 0.0141561919 | n/a | n/a | n/a | n/a |
| $\gamma_{+-}^{--}$ | -0.0165796276 | 0.0075269817 | n/a | n/a | n/a | n/a |
| $\gamma_{++}^{+-}$ | -0.0180480285 | 0.0081936208 | n/a | n/a | n/a | n/a |
| $\gamma_{+0}^{+-}$ | -0.0237108348 | 0.0107644771 | n/a | n/a | n/a | n/a |
| $\gamma_{+-}^{+-}$ | -0.0224627922 | 0.0101978784 | n/a | n/a | n/a | n/a |
| θ _– /_ | **0.1506278413** | **0.0683835028** | n/a | n/a | n/a | n/a |
| θ _/ –_ | **-0.2415187007** | **0.1096470255** | n/a | n/a | n/a | n/a |
| β_0_ | -0.0040105716 | 0.0018207586 | n/a | n/a | n/a | n/a |
| ζ_0_ | -0.0057999737 | 0.0026331289 | n/a | n/a | n/a | n/a |
| ω | -0.0205755847 | 0.0093411055 | n/a | n/a | n/a | n/a |
| ω_b_ | -0.0179604689 | 0.0081538696 | n/a | n/a | n/a | n/a |
| ω_v_ | -0.0242031915 | 0.010988002 | n/a | n/a | n/a | n/a |
| α | 0.0734546646 | 0.0333476682 | n/a | n/a | n/a | n/a |

Indonesia, high TB, no AIDS:

|  | I ^– –^ | I ^– –^ (norm) | I ^+ –^ | I ^+ –^ (norm) | I ^+ +^ | I ^+ +^ (norm) |
| --- | --- | --- | --- | --- | --- | --- |
| c_1_ | -0.0033419175 | 0.0139987899 | n/a | n/a | n/a | n/a |
| h_a_ | -0.0000711605 | 0.0002980806 | n/a | n/a | n/a | n/a |
| h_h_ | -0.0034041678 | 0.0142595467 | n/a | n/a | n/a | n/a |
| h_tb_ | -0.002788939 | 0.0116824461 | n/a | n/a | n/a | n/a |
| d_tb_ | -0.0031694569 | 0.0132763782 | n/a | n/a | n/a | n/a |
| i_tb_ | 0.0019622357 | 0.0082195101 | n/a | n/a | n/a | n/a |
| ρ^– –^ | -0.0052981889 | 0.0221933164 | n/a | n/a | n/a | n/a |
| ρ^+ –^ | 0.0021977589 | 0.0092060814 | n/a | n/a | n/a | n/a |
| ρ^++^ | 0.0056853213 | 0.0238149558 | n/a | n/a | n/a | n/a |
| $\gamma_{++}^{--}$ | **0.0488380661** | **0.2045753129** | n/a | n/a | n/a | n/a |
| $\gamma_{+0}^{--}$ | -0.0001188928 | 0.0004980241 | n/a | n/a | n/a | n/a |
| $\gamma_{+-}^{--}$ | **-0.0528823793** | **0.2215163325** | n/a | n/a | n/a | n/a |
| $\gamma_{++}^{+-}$ | -0.0016915256 | 0.0070855462 | n/a | n/a | n/a | n/a |
| $\gamma_{+0}^{+-}$ | -0.002095635 | 0.0087782998 | n/a | n/a | n/a | n/a |
| $\gamma_{+-}^{+-}$ | -0.0048019924 | 0.0201148238 | n/a | n/a | n/a | n/a |
| θ _– /_ | 0.0028139286 | 0.0117871238 | n/a | n/a | n/a | n/a |
| θ _/ –_ | -0.0043350989 | 0.0181590773 | n/a | n/a | n/a | n/a |
| β_0_ | **0.0492787749** | **0.2064213758** | n/a | n/a | n/a | n/a |
| ζ_0_ | -0.0027311012 | 0.0114401722 | n/a | n/a | n/a | n/a |
| ω | -0.0065076177 | 0.0272594318 | n/a | n/a | n/a | n/a |
| ω_b_ | -0.0069769701 | 0.0292254783 | n/a | n/a | n/a | n/a |
| ω_v_ | -0.0016072171 | 0.0067323905 | n/a | n/a | n/a | n/a |
| α | -0.0053631069 | 0.022465248 | n/a | n/a | n/a | n/a |
