# Supplementary figures and images for: Emergence of antibiotic resistance in immunocompromised host populations: A case study of emerging antibiotic resistant tuberculosis in AIDS patients
Source: PLoS One. 2019 Feb 28;14(2):e0212969. doi: 10.1371/journal.pone.0212969 (PMC6394933; doi:10.1371/journal.pone.0212969)

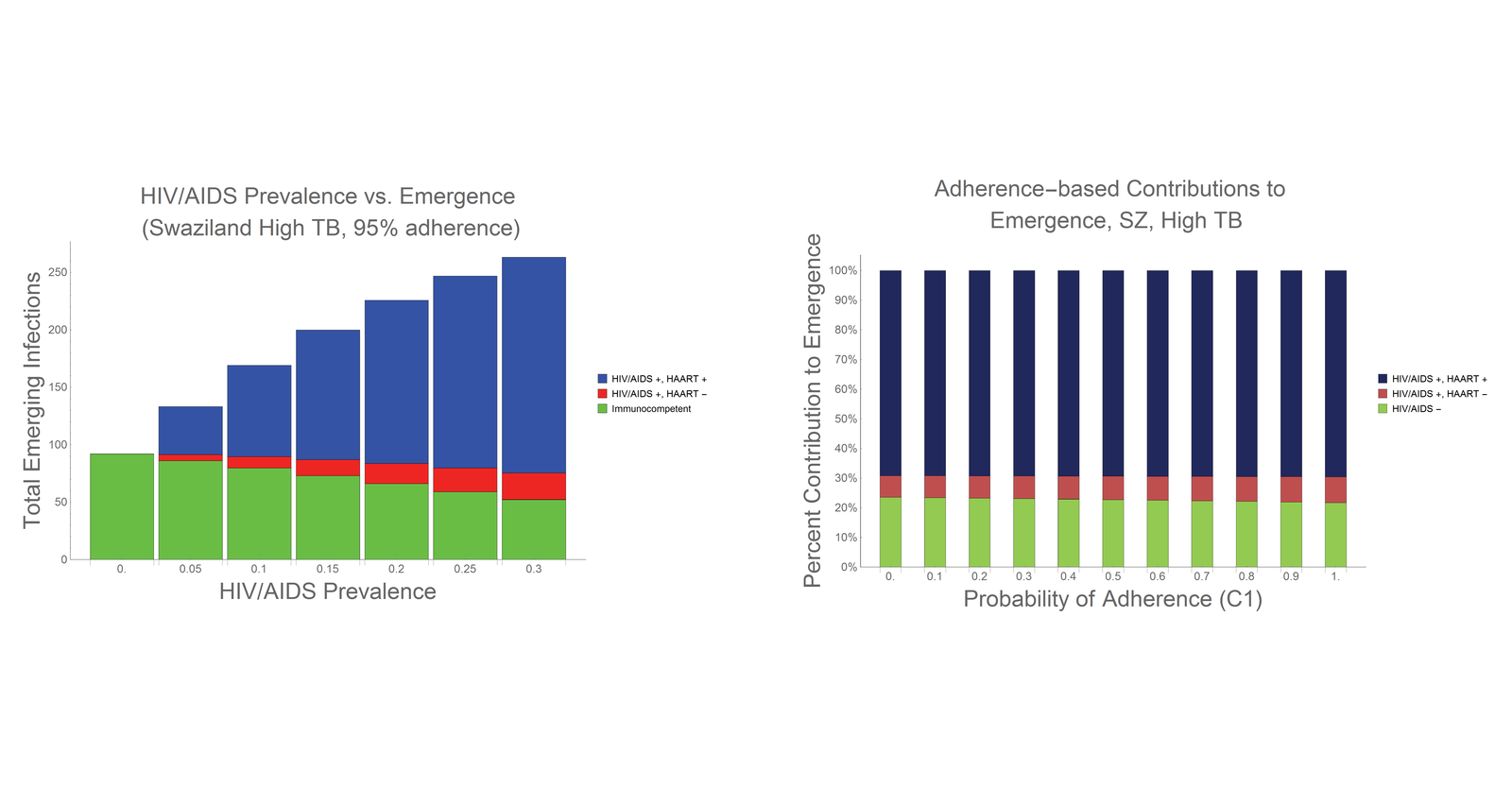

Supplement: S1 Fig — (TIF) [file pone.0212969.s003.tif]

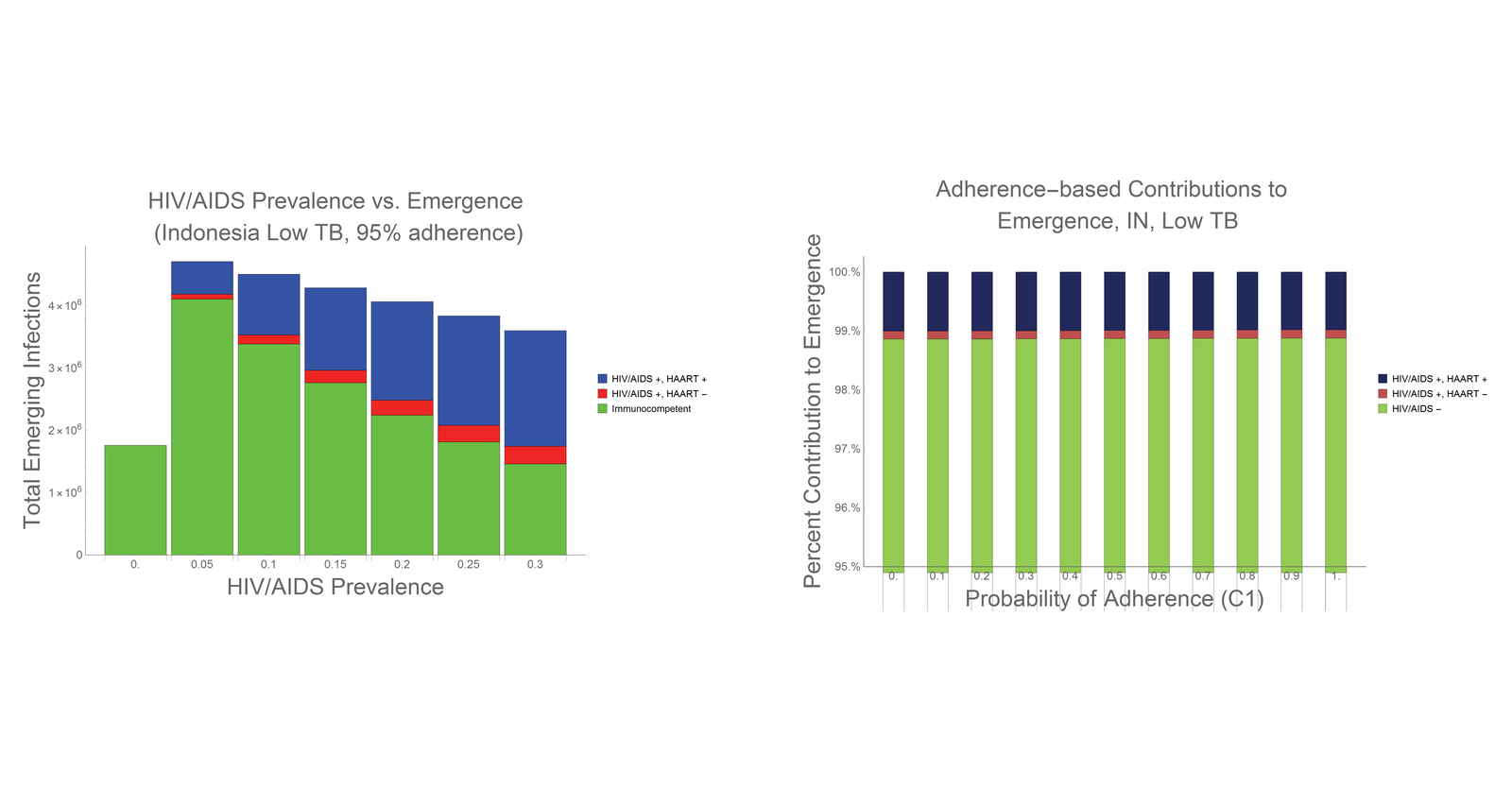

Supplement: S2 Fig — (TIF) [file pone.0212969.s004.tif]

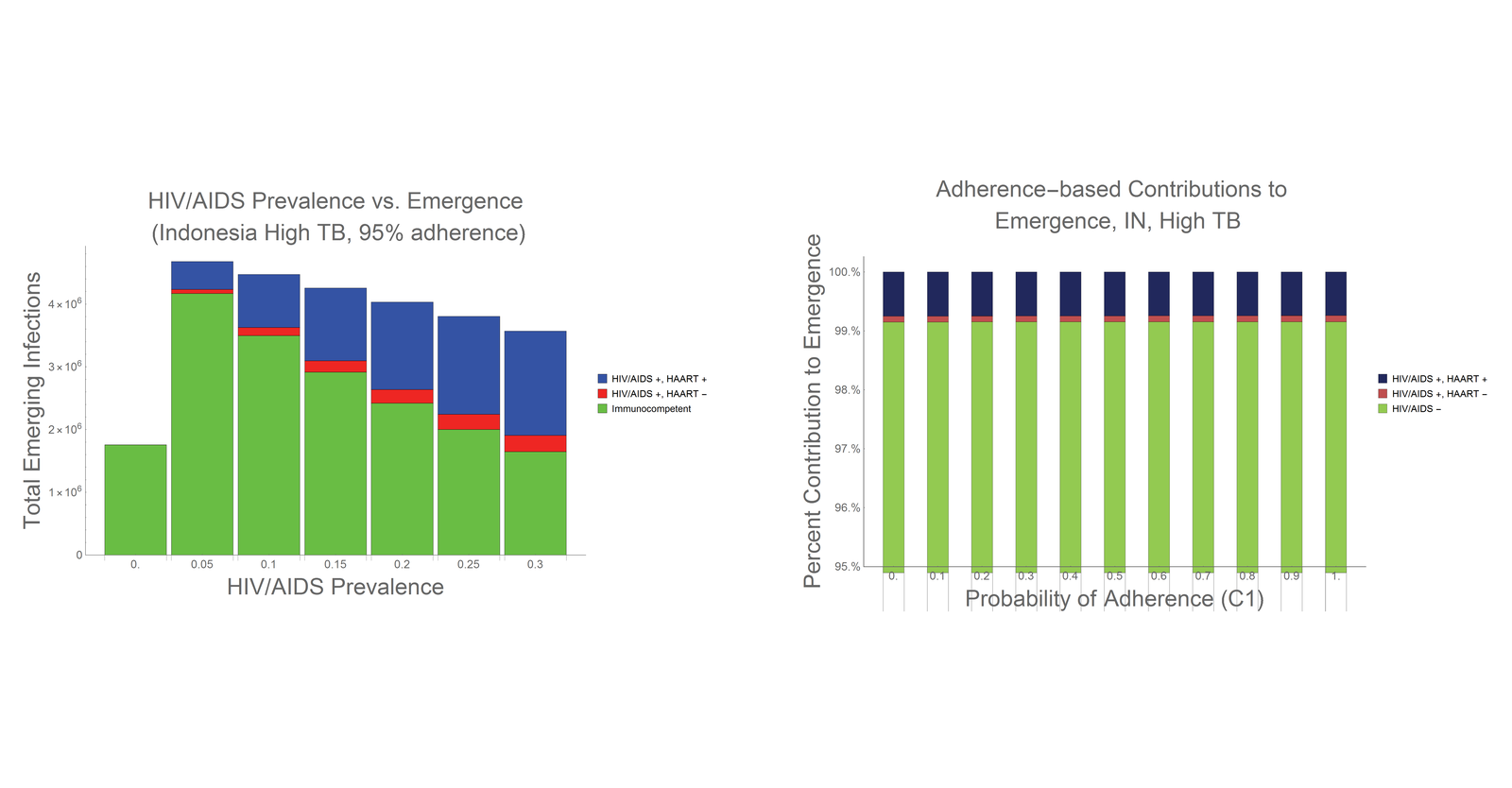

Supplement: S3 Fig — (TIF) [file pone.0212969.s005.tif]
